# Developing ecolabels to encourage sustainable eating in restaurants: A randomized experiment

**DOI:** 10.1371/journal.pone.0335724

**Published:** 2025-10-30

**Authors:** Cristina J. Y. Lee, Joshua Petimar, Amanda B. Zeitlin, Caroline Collis, Lauren Cleveland, Aviva A. Musicus, Anna H. Grummon

**Affiliations:** 1 Department of Pediatrics, Stanford University School of Medicine, Palo Alto, California, United States of America; 2 Department of Population Medicine, Harvard Pilgrim Health Care Institute, Boston, Massachusetts, United States of America; 3 Department of Nutrition, Harvard T.H. Chan School of Public Health, Boston, Massachusetts, United States of America; 4 Center for Science in the Public Interest, Washington, DC, United States of America; 5 Department of Health Policy, Stanford University School of Medicine, Stanford, California, United States of America; Universita degli Studi del Molise, ITALY

## Abstract

Restaurants are increasingly adopting ecolabels to highlight environmentally friendly menu offerings. However, it remains unclear which ecolabel design is the most effective at encouraging consumers to select these items. This study aimed to determine which of 4 common ecolabel formats are perceived as most effective at encouraging selection of environmentally sustainable foods. We conducted an online experiment with 2,169 US adults in August 2023. Participants were randomized to 1 of 5 label formats, including a control format (e.g., QR code) and 4 ecolabel formats: text-plus-icon, text-only, icon-only, or numeric ecolabels. Participants viewed 3 label variations of their randomly assigned label format. Participants rated each label variation on perceived effectiveness for encouraging environmentally sustainable food choices (primary outcome) and other label reactions (e.g., thinking about environmental impacts; secondary outcomes) on 5-point scales. All ecolabels were perceived as more effective at encouraging environmentally sustainable food choices than the control labels (range of average differential effects [ADEs]=.26 to .82, *p*s < .001). Among ecolabels, the text-plus-icon ecolabels were perceived as the most effective (mean = 3.17), followed by icon-only (mean = 2.95), text-only (mean = 2.93), and numeric (mean = 2.62) ecolabels. A relatively similar pattern emerged for secondary outcomes. Text-plus-icon ecolabels may be the most promising ecolabel format to encourage selection of sustainable foods. Restaurants, third-party certification systems, and policymakers interested in encouraging sustainable food choices in restaurants could consider adopting text-plus-icon ecolabels on restaurant menus, though further testing is needed to determine effects on behavior.

## Introduction

Food systems are an important contributor to climate change, accounting for about one-third of human-caused global greenhouse gas emissions [1,2]. Restaurants are a particularly important component of the US and global food system for two reasons. First, consumers buy a significant portion of their food at restaurants. In 2023, Americans spent 56% of their food budget on foods outside of the home, of which 67% was spent at fast food and full-service restaurants [3]. In total, Americans spent over $975 billion at restaurants in 2022 [4]; globally, this figure is $2.3 trillion [5]. Second, fast food and full service restaurants are a major source of foods high in red meat, such as burgers and pizzas [6]. Red meat is responsible for a major share of food-related carbon emissions [7,8], emitting 20 times more greenhouse gases than protein-rich plants such as pulses [1]. For these reasons, interventions that encourage selection of environmentally sustainable foods in restaurants have the potential to reduce dietary carbon emissions.

Many consumers report wanting to consider environmental impacts of their food choices [9,10], but may have challenges doing so at restaurants. For example, consumers may find it difficult to accurately estimate and compare the environmental effects of foods given the extensive range of foods that restaurants offer and the significant variation in environmental impacts across similar foods [1,11,12]. Indeed, consumers tend to underestimate the environmental impacts of foods overall [13,14]. One promising strategy to inform consumers and encourage selection of sustainable foods in restaurants is placing ecolabels on menus to highlight foods with lower environmental impact [15].

Ecolabels are increasingly popular among restaurants and research suggests they hold promise for encouraging more sustainable food choices. For example, previous studies find that front-of-package ecolabels are more effective than neutral or no labels at increasing selection, purchase, and consumption of sustainable foods from grocery stores [16–18]. Other studies have found that ecolabels increase selection of more environmentally sustainable foods from fast food restaurants [19] and cafeterias [20–24], though some studies found null effects [25,26].

What is less well understood is how ecolabels should be designed to maximize their potential to encourage sustainable food selections in restaurants. Different restaurants have adopted a range of ecolabel formats. For example, Panera Bread uses an *icon-only* ecolabel (i.e., without text) showing an interpretive icon developed by the Coolfood Initiative [27]. Other restaurants, such as Sweetgreen, have displayed an interpretive *text-only* ecolabel that read “Earth friendly pick” [28]. Restaurants could also implement interpretive *text-plus-icon* ecolabels, similar to some proposed nutritional labels [29]. Another option, used by Just Salad, is a *numeric* ecolabel displaying the amount of carbon emissions associated with the production of a given menu item [30]. However, it remains unknown which ecolabel format is the most effective at restaurants, as most prior studies of ecolabels have tested only one or two ecolabel formats rather than comparing all popular ecolabel formats against each other [19,23,26,31].

To address this gap, we aimed to test whether ecolabels were perceived as more effective in encouraging consumers to choose environmentally sustainable food options than neutral control labels (i.e., labels unrelated to sustainability) and to identify which ecolabel format (e.g., icon-only, text-plus-icon) was perceived as most effective. We focused on perceived message effectiveness (i.e., the extent to which consumers perceived the labels would encourage them to choose more sustainable foods) as the primary outcome because it is predictive of actual message effectiveness and sensitive to differences between similar messages and is therefore a useful measure in message development studies like this one [32,33]. We also explored whether young adults react differently to ecolabels compared to older adults, given that young adults are especially concerned about environmental sustainability [34,35], and whether participants with varying educational level and party identification respond differently to ecolabels since these are known predictors of climate change awareness and attitudes [36,37]. Findings from the present study could inform the development of ecolabels that are more likely to encourage sustainable eating, which can then be evaluated in subsequent randomized trials with behavioral outcomes.

## Methods

### Participants

We recruited a national convenience sample of 2,169 US adults using the online survey platform CloudResearch Connect. Participants were recruited on August 7^th^, 2023. Participants were eligible if they were 18 years or older and lived in the US. To enhance statistical power to detect moderation between younger and older adults, we oversampled young adults ages 18−29 years such that they comprised approximately half of the sample. The Stanford University (#69580) and Harvard Pilgrim Health Care (#2000970-2) Institutional Review Boards approved the study. We pre-registered the study design and analysis plan before collecting data on ClinicalTrials.gov (NCT # NCT05953246) and AsPredicted.org (https://aspredicted.org/blind.php?x=7L4_TR2).

### Procedures and measures

#### Primary experiment.

**Fig 1:**
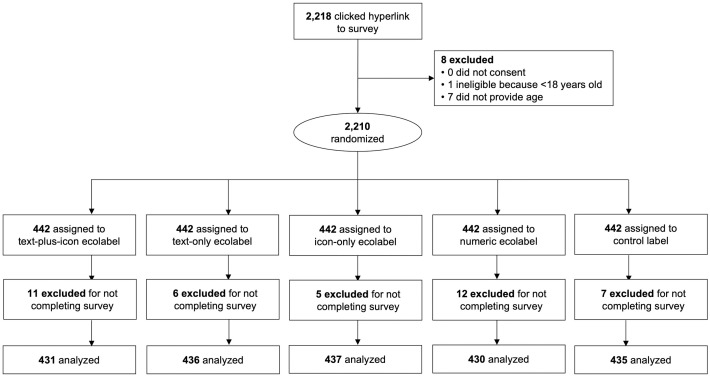
CONSORT flow diagram. Note. Flow diagram shows participant enrollment, exclusion, randomization, and allocation into five experimental conditions.

Participants provided written informed consent via a Qualtrics survey, where they reviewed written consent information and indicated consent by clicking “I consent”. Participants then completed an online survey programmed in Qualtrics. The study consisted of three experimental tasks. The primary experiment tested participants’ reactions to different label *formats* using a 5-condition between-subjects design. Participants were randomized using a simple allocation ratio to 1 of 5 labeling conditions: 1) interpretive text-plus-icon ecolabel (hereafter, text-plus-icon), 2) interpretive text-only ecolabel (text-only), 3) interpretive icon-only ecolabel (icon-only), 4) numeric text-only ecolabel (numeric), or 5) control label. In each condition, participants viewed 3 label variations of their randomly assigned format one at a time and in random order. Each label variation was displayed both on its own and in the context of a menu excerpt at the same time (described below). Participants rated each label variation on perceived effectiveness and secondary outcomes. Fig 1 shows the CONSORT diagram.

**Fig 2:**
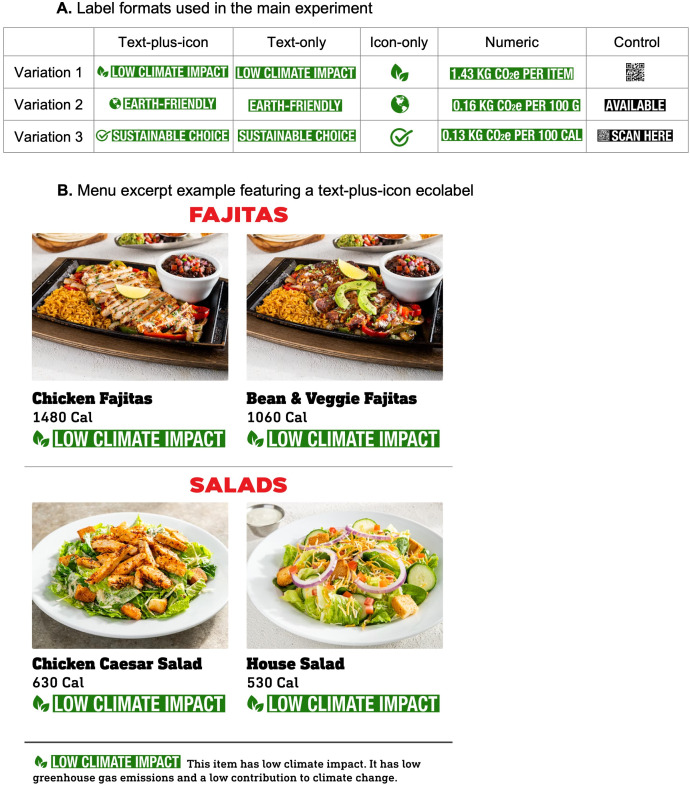
Label formats and menu excerpt example tested in the primary experiment. Note. Figure shows all label format variations tested in the primary experiment. Participants also viewed menu excerpts, such as the example above, each displaying a label format variation of their randomly assigned condition.

We developed 3 label variations for each of 5 label formats, for a total of 15 labels, based on best practices for label design [38,39]. The ecolabels focused on carbon emissions because carbon emissions are the most extensively documented environmental impact of the food system [40] (Fig 2A). The 3 text-only ecolabels displayed interpretive text to indicate the menu item was more sustainable. These labels read “low climate impact”, “earth-friendly”, and “sustainable choice”. The 3 icon-only ecolabels showed an interpretive icon to indicate the menu item was more sustainable. These labels displayed green leaves, a globe, or a check mark. The 3 text-plus-icon ecolabels were a combination of these text-only and icon-only ecolabels. The 3 numeric ecolabels displayed the amount of greenhouse gas emissions in CO_2_e per item, per 100 grams, or per 100 calories (see S1 Supplemental Methods for the carbon emissions calculation methodology). The 3 control labels displayed a QR code, an “available here” text, or a QR code with a “scan here” text.

We then created 15 menu excerpts, each featuring a label variation. Menu excerpts displayed 4 entrees from a popular U.S. full-service restaurant chain. Similar to a prior study [19], we selected entrees without red meat and with lower carbon emissions (compared to entrees with red meat) because they would likely qualify for ecolabels in the real world: chicken fajitas, veggie fajitas, a house salad, and a chicken Caesar salad. Menu excerpts also included an explanatory text that briefly described each label. Fig 2B displays an example of the menu excerpt with a text-plus-icon label.

The primary outcome was perceived message effectiveness, assessed with two items adapted from previous studies [32,33]. One item measured perceived encouragement (“how much does this label make you want to choose items that are more environmentally sustainable?”) and the other item measured appeal (“how much does this label make eating items with this label seem appealing to you?”). Response options for both items ranged from “not at all” (coded as 1) to “a great deal” (coded as 5). We chose perceived message effectiveness as our primary outcome because this measure is sensitive and predictive of long-term change in behavior [32,33]. Responses from the two questions were averaged (Spearman-Brown Prophesy Reliability Estimate = .82).

Four secondary outcomes were measured with one item each. These outcomes were thinking about environmental impacts of foods (“how much does this label make you think about the environmental impacts of your food choices?”), anticipated social interactions with others (“how likely are you to talk about this label with others in the next week?”), attention to labels (“how much does this label grab your attention?”), and believability of labels ("how believable is this label?"). We assessed thinking about environmental impacts of food, anticipated social interactions, and attention to labels because prior research indicates that these constructs may explain the impact of labels on behavior [41–43]. Therefore, labels that lead to larger changes in these constructs may be more promising for encouraging behavior change. We assessed believability of labels because persuasive communications theories posit that messages that are more believable will be more effective [44]. Response options for secondary outcomes ranged from 1 (low value) to 5 (high value). Survey questions are shown in S1 Table.

#### Secondary experiments.

Participants also completed 2 additional within-subjects experiments to provide more insight into optimal design of 2 important elements of ecolabels: text variations and icon variations (S1 Fig). We studied text variations and icon variations because restaurants interested in using ecolabels would need to decide what text and icons to use in their labels, but limited research has compared different text and icons to one another. To gather insight on text variations, participants viewed 6 text-only ecolabels in random order and rated each on perceived message effectiveness using the perceived encouragement item described above. The 6 labels varied in the words shown in the label (“low climate impact”, “earth-friendly”, “sustainable choice”, “environmentally-friendly”, “climate-friendly”, and “low carbon”). To gather insight on icon variations, participants viewed 4 icon-only ecolabels in random order and rated each on perceived message effectiveness, again using the perceived encouragement item described above. The 4 labels varied in the icon design (i.e., a check mark, leaves, a globe, and the Coolfood badge in use at the time of the study [45]). Participants completed these 2 secondary experiments in random order after completing the primary experiment.

### Analysis

Power analysis estimated that a sample size of 2,100 participants (420 per condition) was necessary to detect a small effect size of standardized mean differences between conditions of f = .08 (equivalent to d = .16) or larger with 90% power. To our knowledge, no prior studies have examined perceived message effectiveness of different ecolabel formats, so we selected this effect size based on prior studies evaluating different nutrition label designs [46–50]. We assumed an alpha of .05, and a correlation of .85 among 3 repeated measures (a conservative estimate based on prior studies [46,51]). We aimed to recruit ~2,150 participants to account for potential missing data.

Analyses of the primary experiment on label formats used linear mixed effects regression, regressing outcomes on indicator variables for label formats and excluding the control condition as the referent. The intercept was treated as random to account for repeated measures within participants. We used the models to estimate means and average differential effects (ADEs, i.e., differences in predicted means between conditions) for comparisons between each ecolabel vs. control label and each ecolabel vs. one another. Given the exploratory nature of this study, we did not adjust for multiple comparisons.

We assessed whether the impact of label format on perceived message effectiveness differed by age (younger vs. older adults (18–29 vs. 30+)), education level, or political affiliation (the last two tests were not pre-registered). For each of these analyses, we added to the primary models the moderator and interaction terms between label formats and the moderator. We assessed the joint significance of the interaction terms using a Wald test.

We also used linear mixed effects regression for the two secondary experiments to examine responses to text variations and icon variations. We ran two separate models: one for the text variations experiment and another for the icon variations experiment. Each model regressed the primary outcome on indicator variables for each label variation (i.e., text variation or icon variation), treating the intercept as random. We conducted pairwise comparisons among the text variations and among the icon variations. We did not adjust for multiple comparisons due to the exploratory nature of the analyses. Analyses were conducted in Stata MP version 18 in 2023-2024 using two-tailed tests with an alpha of .05.

## Results

About half of participants (49%) were young adults (ages 18–29), in line with our recruitment plans (Table 1). About half (52%) of the sample identified as men, 46% as women, and 2% as non-binary or another gender. Most participants (71%) were White, 12% were Black or African American, 11% were Hispanic, and 9% were Asian or Pacific Islander. About one-third (34%) of the sample had educational attainment of some college or less and 36% had an annual household income of less than $50,000. About half (56%) of participants identified as Democrat.

**Table 1:** Participant characteristics by label condition (n = 2,169).

Characteristic	Text-plus-icon (n = 431)	Text-only (n = 436)	Icon-only (n = 437)	Numeric (n = 430)	Control (n = 435)
Age					
18–29 years	227 (53%)	210 (48%)	213 (49%)	216 (50%)	206 (47%)
30–44 years	127 (29%)	144 (33%)	134 (31%)	127 (30%)	159 (37%)
45–59 years	56 (13%)	55 (13%)	58 (13%)	67 (16%)	44 (10%)
60 years or older	21 (5%)	27 (6%)	32 (7%)	20 (5%)	26 (6%)
Gender					
Female	214 (50%)	210 (48%)	192 (44%)	197 (46%)	190 (44%)
Male	208 (48%)	218 (50%)	239 (55%)	217 (50%)	236 (54%)
Non-binary or another gender	9 (2%)	8 (2%)	6 (1%)	16 (4%)	9 (2%)
Race					
White	302 (70%)	315 (72%)	313 (72%)	304 (71%)	297 (68%)
Black or African American	58 (13%)	52 (12%)	51 (12%)	53 (12%)	52 (12%)
American Indian or Alaska Native	8 (2%)	5 (1%)	7 (2%)	5 (1%)	4 (1%)
Asian or Pacific Islander	37 (9%)	37 (8%)	36 (8%)	47 (11%)	47 (11%)
Other or Multiracial	26 (6%)	27 (6%)	30 (7%)	21 (5%)	35 (8%)
Latino(a) or Hispanic	45 (10%)	47 (11%)	44 (10%)	47 (11%)	53 (12%)
Education					
High school diploma or less	52 (12%)	52 (12%)	69 (16%)	60 (14%)	56 (13%)
Some college	87 (20%)	86 (20%)	89 (20%)	85 (20%)	95 (22%)
College graduate or Associate’s degree	236 (55%)	223 (51%)	217 (50%)	223 (52%)	216 (50%)
Graduate degree	56 (13%)	75 (17%)	62 (14%)	62 (14%)	68 (16%)
Household income, annual					
$0 to $24,999	51 (12%)	48 (11%)	63 (14%)	63 (15%)	68 (16%)
$25,000 to $49,999	92 (21%)	99 (23%)	105 (24%)	99 (23%)	101 (23%)
$50,000 to $74,999	104 (24%)	95 (22%)	90 (21%)	89 (21%)	77 (18%)
$75,000 or more	184 (43%)	194 (44%)	178 (41%)	179 (42%)	189 (43%)
Household size					
1-2	204 (47%)	211 (48%)	218 (50%)	196 (46%)	197 (45%)
3-4	174 (40%)	183 (42%)	178 (41%)	179 (42%)	188 (43%)
5 or more	53 (12%)	42 (10%)	41 (9%)	55 (13%)	50 (11%)
Number of children					
0	292 (68%)	290 (67%)	308 (70%)	283 (66%)	286 (66%)
1-2	114 (26%)	132 (30%)	117 (27%)	126 (29%)	127 (29%)
3 or more	25 (6%)	14 (3%)	12 (3%)	21 (5%)	22 (5%)
Political party identification					
Democrat	265 (61%)	251 (58%)	229 (52%)	233 (54%)	246 (57%)
Republican	91 (21%)	105 (24%)	103 (24%)	114 (27%)	105 (24%)
Independent	66 (15%)	74 (17%)	87 (20%)	79 (18%)	72 (17%)
Other	9 (2%)	6 (1%)	18 (4%)	4 (1%)	12 (3%)

Note: Percentages may not add to total due to rounding.

### Ecolabel formats experiment

#### Perceived message effectiveness.

**Fig 3:**
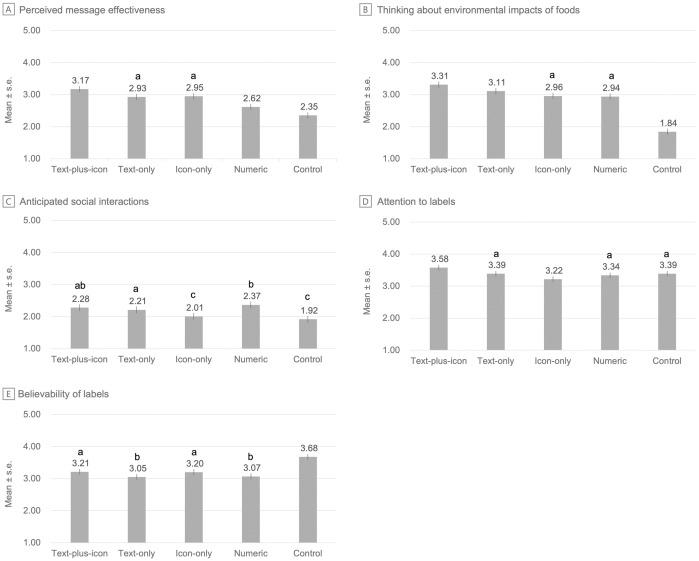
Perceived message effectiveness, thinking about environmental impacts of foods, anticipated social interactions, attention to labels, and believability of labels, by label format. Note. s.e. = standard error. Means with the same superscript letters are *not* statistically significantly different from one another at p < .05.

All four ecolabel formats (i.e., text-plus-icon, text-only, icon-only, and numeric) were perceived as more effective than the control labels (range of average differential effects [ADEs]=.26 to .82, *p* for each ecolabel vs. control < .001, S2 Table). The text-plus-icon ecolabels were perceived as the most effective (mean [standard error (SE)] = 3.17 [.05]), followed by the icon-only (2.95 [.05]), the text-only (2.93 [.05]), the numeric (2.62 [.05]), and the control labels (2.35 [.05], Fig 3). There were several differences in perceived message effectiveness between the ecolabels. The text-plus-icon ecolabels were perceived as more effective than the text-only (ADE = .24 [95% CI = .11 to .37]), the icon-only (ADE = .22 [95% CI = .09 to .35]), and the numeric ecolabels (ADE = .55 [95% CI = .42 to .69], *p*s for comparisons<=.001). Next, the text-only ecolabels were perceived similarly as effective as the icon-only (ADE = −.02 [95% CI = −.15 to .11], *p* = .77), but more effective than numeric ecolabels (ADE = .31 [95% CI = .18 to .44], *p* < .001). Lastly, the icon-only ecolabels were perceived as more effective than numeric ecolabels (ADE = .33 [95% CI = .20 to .46], *p* < .001).

**Fig 4:**
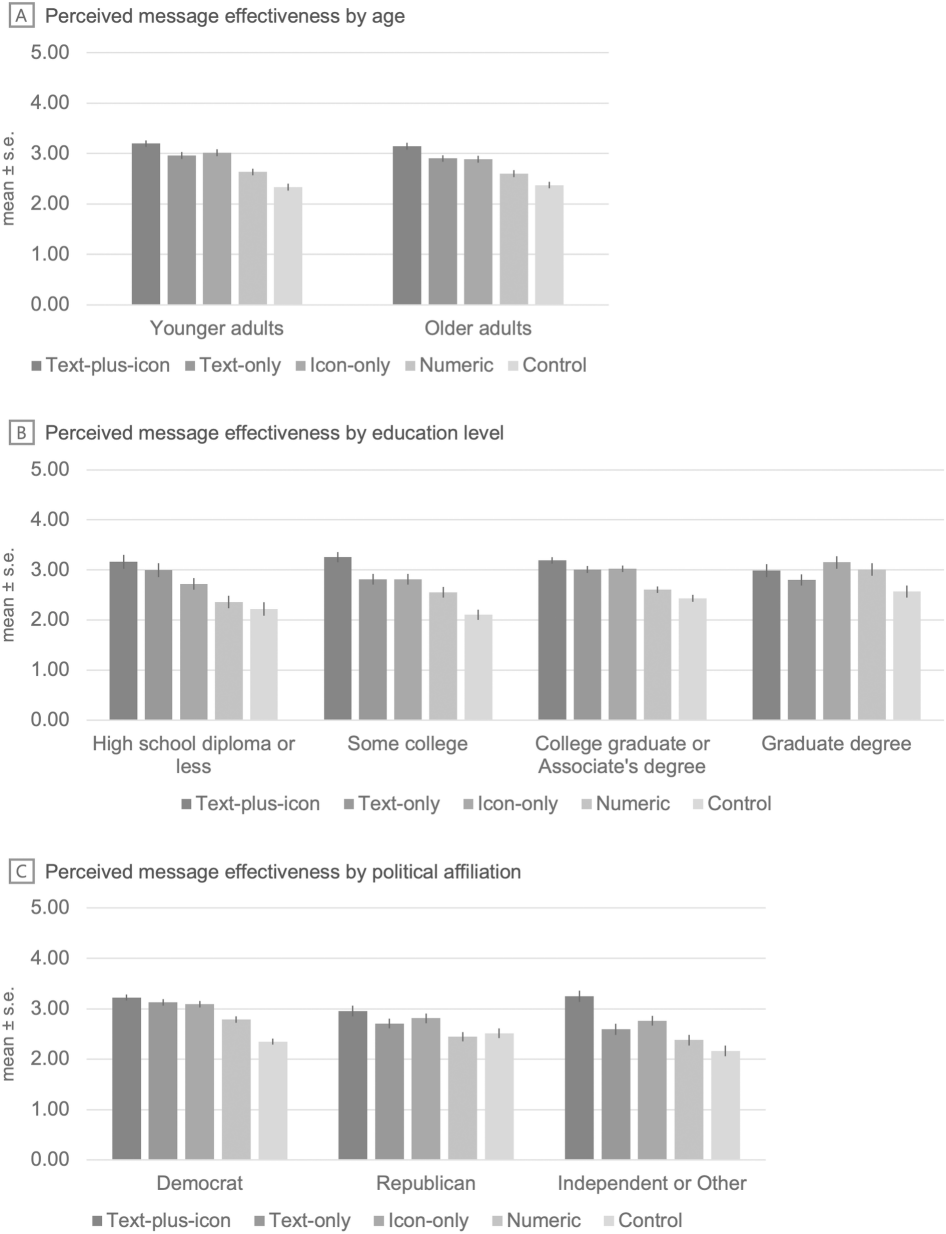
Effects of label formats on perceived message effectiveness by (A) age, (B) education level, and (C) political affiliation. Note. *p* for interaction **(A)** =.79, *p* for interaction **(B)** =.007, and *p* for interaction **(C)** =.001. ADEs are listed in S3 Table.

In moderation analyses, the effects of label format on perceived message effectiveness were not moderated by age (younger vs. older adults) (*p* for joint significance of interaction terms = .79), but were moderated by education level and political affiliation (*p*s for interaction <=.007, Fig 4, S3 Table). For education level, the effects of the different ecolabels vs. the control differed among participants with graduate degrees compared to those with lower educational attainment. For example, the numeric ecolabels were perceived as more effective among participants with graduate degrees compared to those with other education levels. For political affiliation, effects of the different ecolabels vs. the control differed among participants identifying as Democrat (often associated with liberal or progressive ideologies) compared to those identifying as Republican (often associated with conservative ideologies) or as Independent or Other. For instance, the text-only ecolabels were perceived as more effective among participants identifying as Democrats vs. those identifying as Republican or as Independent or Other.

#### Secondary outcomes.

Ecolabels generally performed better than the control labels on 3 out of 4 secondary outcomes: thinking about environmental impacts of foods, anticipated social interactions, and attention to labels (Fig 3). Ecolabels, however, did not outperform control labels on believability.

When comparing among ecolabel formats, the text-plus-icon consistently outperformed other formats on the secondary outcomes, but otherwise, which ecolabel formats outperformed the others differed depending on the secondary outcome (S2 Table). For example, all four ecolabel formats led to higher ratings of thinking about the environmental impacts of foods than the control labels (*p* for each ecolabel vs. control < .001). The pattern of results was similar to that of perceived message effectiveness: among ecolabels, the text-plus-icon ecolabels led to the highest ratings of thinking about the environmental impacts of food (mean [SE] = 3.31 [.05]), followed by the text-only (3.11 [.05]), the icon-only (2.96 [.05]), and the numeric ecolabels (2.94 [.05]). Like perceived message effectiveness, the text-plus-icon ecolabels led to higher ratings of thinking about the environmental impacts of foods than the text-only (ADE = .20 [95% CI = .07 to .34]), the icon-only (ADE = .35 [95% CI = .22 to .49]), and the numeric ecolabels (ADE = .37 [95% CI = .24 to .51], *p*s for comparisons<=.004). The text-only ecolabels led to higher ratings of thinking about the environmental impacts of foods than both the icon-only (ADE = .15 [95% CI = .02 to .29], *p* = .03) and the numeric ecolabels (ADE = .17 [95% CI = .04 to .31], *p* = .01). The icon-only ecolabels led to similar ratings of thinking about environmental impacts of food as the numeric ecolabels (*p* = .76).

Three of four ecolabel formats (text-plus-icon, text-only, numeric, but not icon-only) led to higher ratings of anticipated social interactions than the control labels (*p* for these ecolabels vs. control < .001). The pattern of results differed from that of perceived message effectiveness and thinking about the environmental impacts of foods. In this case, the numeric ecolabels led to the highest ratings of anticipated social interactions (mean [SE]=2.37 [.05]) followed by the text-plus-icon (2.28 [.05]), the text-only (2.21 [.05]), and the icon-only ecolabels (2.01 [.05]), though not all differences between ecolabels were statistically significant. Specifically, the text-plus-icon ecolabels led to similar ratings of anticipated social interactions as the text-only ecolabels (*p* = .37), higher ratings of anticipated social interactions than the icon-only ecolabels (ADE = .27 [95% CI = .12 to .42], *p* < .001), and similar ratings of anticipated social interactions as the numeric ecolabels (*p* = .26). Next, the text-only ecolabels led to higher ratings of anticipated social interactions compared to the icon-only ecolabels (ADE = .20 [95% CI = .05 to .35], *p* = .01), but lower ratings of anticipated social interactions than the numeric ecolabels (ADE = −.16 [95% CI = −.31 to −.01], *p* = .04). The icon-only ecolabels led to lower ratings of anticipated social interactions than the numeric ecolabels (ADE = −.36 [95% CI = −.51 to −.21], *p* < .001).

Only one of four ecolabel formats (text-plus-icon) elicited more attention than the control labels (ADE = .19 [95% CI = .07 to .30], p = .002). Among ecolabels, the text-plus-icon ecolabels elicited the most attention (mean [SE]) = 3.58 [.04]), followed by the text-only (3.39 [.04]), the numeric (3.34 [.04]), and the icon-only ecolabels (3.22 [.04]). The text-plus-icon ecolabels elicited more attention than the text-only (ADE = .19 [95% CI = .07 to .31]), the icon-only (ADE = .36 [95% CI = .24 to .47]), and the numeric ecolabels (ADE = .24 [95% CI = .12 to .36], *p*s for comparisons<=.002).

All four ecolabel formats were perceived as less believable than the control labels (*p* for each ecolabel vs. control < .001). Among ecolabels, the text-plus-icon ecolabels were perceived as the most believable (mean [SE] = 3.21 [.04]), followed by the icon-only (3.20 [.04]), the numeric (3.07 [.04]), and the text-only ecolabels (3.05 [.04]), though not all differences between ecolabels were significant. The text-plus-icon ecolabels were perceived as more believable than the text-only ecolabels (ADE = .16 [95% CI = .03 to .28], *p* = .01), similarly believable as the icon-only ecolabels (p = .90), and more believable than the numeric ecolabels (ADE = .14 [95% CI = .02 to .26], *p* = .03). The text-only ecolabels were perceived as less believable than icon-only ecolabels (ADE = −.15 [95% CI = −.27 to −.03], *p* = .02) and similarly believable as numeric ecolabels (*p* = .78). Finally, icon-only ecolabels were perceived as more believable than numeric ecolabels (ADE = .13 [95% CI = .01 to .26], *p* = .04).

### Ecolabel text and icon variations

**Fig 5:**
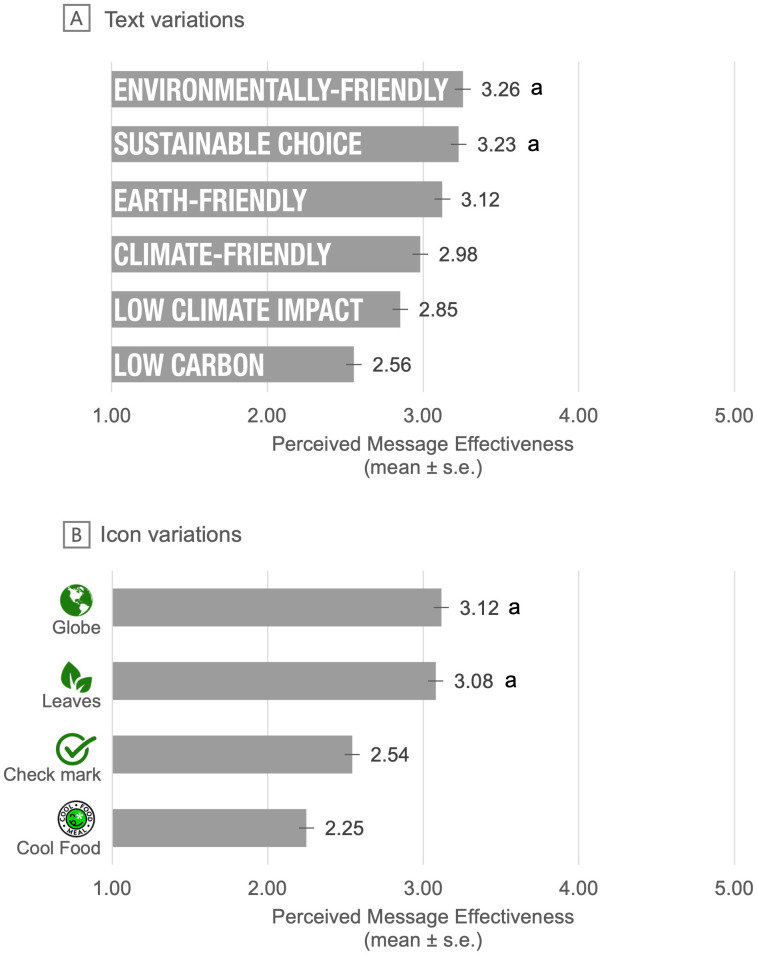
Perceived message effectiveness by (A) text variations and (B) icon variations. Note. s.e. = standard error. Means with the same superscript letters are *not* statistically significantly different from one another at p < .05.

Among the six text variations of text-only ecolabels, “environmentally-friendly” (mean [SE] = 3.26 [.03]) was perceived as the most effective, followed by “sustainable choice” (3.23 [.03]), “earth-friendly” (3.12 [.03]), “climate-friendly” (2.98 [.03]), “low climate impact” (2.85 [.03]), and “low carbon” ecolabels (2.56 [.03], Fig 5). When comparing these variations to one another, “environmentally-friendly” and “sustainable choice” were perceived as similarly effective (p = .23) and both were perceived as more effective than the other four text variations (*p*s for comparisons < .001).

Among the four icon variations, the globe was perceived as the most effective (mean [SE] =3.12 [.02]), followed by the leaves (3.08 [.02]), the check mark (2.54 [.02]), and the Coolfood badge (2.25 [.02]). When comparing these variations to one another, the globe and the leaves icons were perceived as similarly effective (p = .16), and both were perceived as more effective than the check mark and the Coolfood badge (*p*s for comparisons < .001).

## Discussion

This randomized experiment found that all four ecolabel formats (text-plus-icon, text-only, icon-only, and numeric) were perceived as more effective than control labels for encouraging selection of environmentally sustainable restaurant menu options. Among the ecolabels, the text-plus-icon ecolabel ranked highest on perceived message effectiveness and highest or tied for highest on ratings of thinking about environmental impacts of foods, anticipated social interactions, and attention to labels. The text-plus-icon was also perceived as among the most believable ecolabel formats, although all ecolabel formats were perceived as less believable than the control labels. Because perceived message effectiveness is predictive of actual message effectiveness [33], these findings suggest that the text-plus-icon ecolabel is a promising label format to encourage consumers to choose sustainable foods at restaurants, though future studies assessing actual food choice will be needed.

In general, the interpretive ecolabel formats (text-plus-icon, text-only, and icon-only) performed better than the numeric ecolabels. Specifically, the interpretive ecolabels were perceived as more effective and elicited higher ratings of thinking about environmental impacts of foods than the numeric ecolabels. These results are in line with research on front-of-package nutrition labeling schemes, which finds that interpretive labels are more effective at encouraging selection of healthier products than numeric calorie labels [47–49,52].

The text-plus-icon ecolabel format performed the best or tied for best on all outcomes among interpretive ecolabels. While a systematic review of ecolabels for pre-packaged products (rather than restaurant menus) did not identify a particular ecolabel format as the most effective [16], our findings are consistent with studies of nutrition and alcohol warning labels that indicate that text-plus-icon labels are more effective than other label formats [48,49,52,53]. Our study also found that the text variations “environmentally-friendly” and “sustainable choice” and the globe and leaves icon variations were perceived as most effective. A prior study showed that text-plus-icon ecolabels increased selection of sustainable menu items compared to neutral QR code labels [19]; our results suggest that benefits could be even larger when incorporating these text and icon variations into the text-plus-icon ecolabel format. Restaurants can consider using these label element variations when designing text-plus-icon ecolabels for their menus.

The numeric ecolabels were perceived as one of the least effective formats overall, though they were perceived as more effective among participants with graduate degrees compared to those with other education levels. Additionally, the numeric ecolabels elicited high anticipated social interactions. Most consumers – perhaps especially those with lower educational attainment – might have difficulty interpreting the numeric information presented in the numeric ecolabels, leading them to perceive these labels as less effective. At the same time, these consumers might find the numeric ecolabel interesting given its novel information about carbon emissions, prompting them to anticipate talking about the numeric ecolabel with others.

Although all ecolabel formats were perceived as more effective at encouraging selection of environmentally sustainable options than the control labels, they were also perceived as less believable than the control labels. A similar pattern of findings was observed in studies examining alcohol warning labels. Those studies found that warning labels about cancer were perceived as more novel and less believable compared to more familiar warning label topics such as liver disease. Moreover, cancer warnings were among the most effective topics for making participants consider drinking less alcohol, despite having low believability [54,55]. This suggests that novel label topics may elicit less believability given that consumers are less informed about such topics, but these topics can still encourage consumers to change their behavior. Efforts to educate and inform consumers about ecolabels and carbon emissions of foods may increase believability of ecolabels.

Participants might have also found the ecolabels less believable than the control labels because of an underlying skepticism toward corporate efforts to market products as “sustainable.” Studies have found that companies’ use of misleading marketing practices like “greenwashing” (i.e., deceptive or false claims about the environmental impacts of products [56]) is associated with higher consumer skepticism of the validity of environmental claims [57–59]. Consumers report they generally mistrust ecolabels because companies often do not provide evidence, verification, and transparency to support their environmental claims [60,61]. To protect consumers from such misleading practices, regulators could increase their oversight of the use of ecolabels. For example, the European Union “Greenwashing Directive” and “Green Claims Directive” proposals require companies to verify environmental claims through independent third-party entities [62,63].

Given the growing demand for climate action from governments, civil society, and businesses [64], coupled with pledges to reduce their carbon footprints [65], restaurants should consider adopting effective ecolabels on their menus. As more restaurants implement ecolabels, a proliferation of formats may cause confusion among consumers [66,67], highlighting the need for standardized ecolabels. Our results suggest text-plus-icon labels are especially promising, and third-party certification systems and policymakers could consider adopting the text-plus-icon ecolabel design as a standard format for restaurant menus.

Strengths of the study included the randomized experimental design, our oversampling of young adults to adequately assess effect moderation by age group, and the use of realistic menu items from a popular restaurant in the US. This study also had limitations. First, we adapted the original perceived message effectiveness scale to better reflect the behavior in question. We adapted two out of the three items from the original scale (i.e., discouragement and unpleasantness) because the third item (i.e., concern about the health effects of the behavior) was less relevant to ecolabels. Although the items we used were not validated, they showed good reliability. Second, the experiments relied on self-reported outcomes instead of behavioral outcomes, though messages that are perceived as more effective are also more likely to change behavior [32,33]. Our findings could therefore inform future randomized trials evaluating whether evidence-based ecolabels influence behavioral outcomes such as purchases or consumption. Third, we recruited a convenience sample that somewhat differed from the US population, likely due to the oversampling of young adults. Jeong et al. found that although the demographic characteristics of convenience and probability samples differ, experiments conducted with convenience samples yield similar findings as those conducted in nationally representative samples [68]. Fourth, we used a simplified menu with a limited number of items instead of a full menu. The stimuli may have led to larger effect sizes as the labels could have drawn more attention compared to when shown in the context of an entire menu. Fifth, we selected entrée items that did not have red meat and had lower carbon emissions to receive the ecolabels in our study. Although these items are likely to be eligible for ecolabels, restaurants may use different criteria to assign ecolabels than we used. Future studies should identify a standard sustainable food profiling model to support consistent implementation of ecolabels across restaurants. Sixth, consumer interest in sustainability could potentially moderate the effect of the ecolabels, but we did not assess it. However, in a subsequent study [69] assessing the effect of the text-plus-icon ecolabel on restaurant meal selections, interest in sustainability did not moderate the effect of the ecolabel on meal selection. Seventh, we only tested ecolabels focused on carbon emissions; future studies should assess ecolabels that encompass other environmental impacts of food production such as land use, water use, eutrophication, or biodiversity.

## Conclusion

Restaurants have the opportunity to help reduce carbon emissions by encouraging consumption of more sustainable foods. One promising tool to achieve this goal is displaying ecolabels on restaurant menus to alert consumers to more sustainable options. Results from this study indicate a variety of ecolabel formats are perceived as more effective at encouraging consumers to choose environmentally sustainable foods, and that text-plus-icon ecolabels are especially promising. Restaurants, third-party certification systems, and policymakers interested in encouraging sustainable food choices could consider using text-plus-icon ecolabels on restaurant menus, though further testing in field settings is needed to assess effects on behavior.

## Supporting information

S1 Supplemental Methods Estimating carbon emissions for numeric ecolabels. (PDF)

S1 Fig Stimuli tested in the secondary experiments. (PDF)

S1 Table Survey questions. (PDF)

S2 Table Impact of label format on primary and secondary outcomes (n = 2,169). (PDF)

S3 Table Effects of label format on perceived message effectiveness by age, education level, and political affiliation (n = 2,169). (PDF)

S1 File Linear mixed effect regression results of primary and secondary experiments. (PDF)
