# Supplementary material for: Developing ecolabels to encourage sustainable eating in restaurants: A randomized experiment
Source: PLoS One. 2025 Oct 30;20(10):e0335724. doi: 10.1371/journal.pone.0335724 (PMC12574897; doi:10.1371/journal.pone.0335724)
Supplement: S1 Supplemental Methods — (PDF) [file pone.0335724.s001.pdf]

S1 Supplemental Methods. Estimating carbon emissions for numeric ecolabels

Menu items from Chili's were disaggregated into food components and ingredients based on the item description and nutrition facts documentation published by the restaurant. For items that lacked composition information, we used information from comparable items of other restaurant chains. We then assigned USDA Food and Nutrient Database for Dietary Studies (FNDDS) food codes to each food component.

For most of the food components where caloric data was available, but not their weight information, we converted calories into grams by using the FNDDS reference database [1], which provides caloric data per 100g of specific types of foods (restaurant's food component kcal * (100 g / FNDDS reference food kcal)). Next, we linked FNDDS codes with greenhouse gas (GHG) emissions data, expressed in carbon dioxide equivalents, from the Database of Food Recall Impacts on the Environment for Nutrition and Dietary Studies (dataFRIENDS) [2].

Then, we multiplied GHG emissions of each food component with their corresponding weight. To calculate the total carbon emissions per item, we summed the GHG emissions of the food components and ingredients associated with each menu item. To calculate carbon emissions per 100g of a menu item, we divided the total carbon emissions per item by the total weight of each item and multiplied by 100. To calculate the carbon emissions per 100kcal of a menu item, we divided the total carbon emissions per item by the total caloric content of each item and multiplied by 100.

References:

1. U.S. Department of Agriculture, Agricultural Research Service. 2018. USDA Food and Nutrient Database for Dietary Studies 2015-2016. Food Surveys Research Group Home Page, http://www.ars.usda.gov/nea/bhnrc/fsrg

2. Diet, health, and the environment [Internet]. Tulane University Celia Scott Weatherhead School of Public Health and Tropical Medicine. [cited 2024 Dec 10]. Available from: https://sph.tulane.edu/sbps/diet-environmental-impacts
