# Supplementary material for: Developing ecolabels to encourage sustainable eating in restaurants: A randomized experiment
Source: PLoS One. 2025 Oct 30;20(10):e0335724. doi: 10.1371/journal.pone.0335724 (PMC12574897; doi:10.1371/journal.pone.0335724)

S1 Fig. Stimuli tested in the secondary experiments

Panel A. Text variations used in the secondary experiment

LOW CARBON
LOW CLIMATE IMPACT
SUSTAINABLE CHOICE
CLIMATE-FRIENDLY
EARTH-FRIENDLY
ENVIRONMENTALLY-FRIENDLY

Panel B. Icon variations used in the secondary experiment

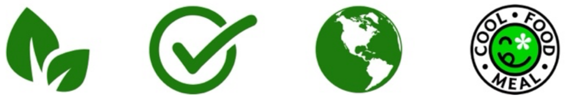

Supplement: S1 Fig — (PDF) [file pone.0335724.s002.pdf]
