# Supplementary material for: Developing ecolabels to encourage sustainable eating in restaurants: A randomized experiment
Source: PLoS One. 2025 Oct 30;20(10):e0335724. doi: 10.1371/journal.pone.0335724 (PMC12574897; doi:10.1371/journal.pone.0335724)
Supplement: S1 Table — (PDF) [file pone.0335724.s003.pdf]

S1 Table. Survey questions

Outcome	Survey question	Response options
Ecolabel formats		
Perceived Message Effectiveness – Encouragement	How much does this label make you want to choose items that are more environmentally sustainable?	1=Not at all<br>2=Very little<br>3=Somewhat<br>4=Quite a bit<br>5=A great deal
Perceived Message Effectiveness – Appealing	How much does this label make eating items with this label seem appealing to you?	1=Not at all<br>2=Very little<br>3=Somewhat<br>4=Quite a bit<br>5=A great deal
Thinking about environmental impacts of foods	How much does this label make you think about the environmental impacts of your food choices?	1=Not at all<br>2=Very little<br>3=Somewhat<br>4=Quite a bit<br>5=A great deal
Anticipated social interactions	How likely are you to talk about this label with others in the next week?	1=Not at all likely<br>2=A little likely<br>3=Somewhat likely<br>4=Very likely<br>5=Extremely likely
Attention to labels	How much does this label grab your attention?	1=Not at all<br>2=Very little<br>3=Somewhat<br>4=Quite a bit<br>5=A great deal
Believability of labels	How believable is this label?	1=Not at all<br>2=Very little<br>3=Somewhat<br>4=Quite a bit<br>5=A great deal
Ecolabel text and icon variations		
Perceived Message Effectiveness – Encouragement	How much does this label make you want to choose environmentally sustainable items?	1=Not at all<br>2=Very little<br>3=Somewhat<br>4=Quite a bit<br>5=A great deal
