# Supplementary material for: Developing ecolabels to encourage sustainable eating in restaurants: A randomized experiment
Source: PLoS One. 2025 Oct 30;20(10):e0335724. doi: 10.1371/journal.pone.0335724 (PMC12574897; doi:10.1371/journal.pone.0335724)
Supplement: S2 Table — (PDF) [file pone.0335724.s004.pdf]

S2 Table. Impact of label format on primary and secondary outcomes (n=2,169)

	**Perceived message effectiveness**			**Thinking about environmental impacts of foods**			**Anticipated social interactions**			**Attention to labels**			**Believability of labels**		
**Pairwise Comparisons**	**ADE**	**95% C.I.**	***p***	**ADE**	**95% C.I.**	***p***	**ADE**	**95% C.I.**	***p***	**ADE**	**95% C.I.**	***p***	**ADE**	**95% C.I.**	***p***
Text-plus-icon x Control	**.82**	**.68, .95**	**<.001**	**1.47**	**1.33, 1.61**	**<.001**	**.36**	**.21, .51**	**<.001**	**.19**	**.07, .30**	**.002**	**-.47**	**-.59, -.34**	**<.001**
Text-only x Control	**.58**	**.44, .71**	**<.001**	**1.27**	**1.13, 1.40**	**<.001**	**.29**	**.14, .44**	**<.001**	-.004	-.12, .11	.95	**-.63**	**-.75, -.50**	**<.001**
Icon-only x Control	**.60**	**.46, .73**	**<.001**	**1.12**	**.98, 1.25**	**<.001**	.09	-.06, .24	.23	**-.17**	**-.29, -.06**	**.004**	**-.48**	**-.60, -.35**	**<.001**
Numeric x Control	**.26**	**.13, .40**	**<.001**	**1.10**	**.96, 1.23**	**<.001**	**.45**	**.30, .60**	**<.001**	-.05	-.17, .06	.38	**-.61**	**-.73, -.48**	**<.001**
Text-plus-icon x Text-only	**.24**	**.11, .37**	**<.001**	**.20**	**.07, .34**	**.004**	.07	-.08, .22	.37	**.19**	**.07, .31**	**.002**	**.16**	**.03, .28**	**.01**
Text-plus-icon x Icon-only	**.22**	**.09, .35**	**.001**	**.35**	**.22, .49**	**<.001**	**.27**	**.12, .42**	**<.001**	**.36**	**.24, .47**	**<.001**	.01	-.12, .13	.90
Text-plus-icon x Numeric	**.55**	**.42, .69**	**<.001**	**.37**	**.24, .51**	**<.001**	-.09	-.24, .06	.26	**.24**	**.12, .36**	**<.001**	**.14**	**.02, .26**	**.03**
Text-only x Icon-only	-.02	-.15, 11	.77	**.15**	**.02, .29**	**.03**	**.20**	**.05, .35**	**.01**	**.17**	**.05, .29**	**.005**	**-.15**	**-.27, -.03**	**.02**
Text-only x Numeric	**.31**	**.18, .44**	**<.001**	**.17**	**.04, .31**	**.01**	**-.16**	**-.31, -.01**	**.04**	.05	-.07, .17	.41	-.02	-.14, .11	.78
Icon-only x Numeric	**.33**	**.20, .46**	**<.001**	.02	-.12, .16	.76	**-.36**	**-.51, -.21**	**<.001**	**-.12**	**-.24, .00**	**.04**	**.13**	**.01, .26**	**.04**

ADE = Average Differential Effect. Response options for outcomes ranged from 1 (low values) to 5 (high values). Statistically significant differences are in bold, $p<.05$.
