# Supplementary material for: Developing ecolabels to encourage sustainable eating in restaurants: A randomized experiment
Source: PLoS One. 2025 Oct 30;20(10):e0335724. doi: 10.1371/journal.pone.0335724 (PMC12574897; doi:10.1371/journal.pone.0335724)
Supplement: S3 Table — (PDF) [file pone.0335724.s005.pdf]

S3 Table. Effects of label format on perceived message effectiveness by age, education level, and political affiliation (*n*=2,169)

Characteristic	Numeric		Icon-only		Text-only		Text-plus-icon		*p* for interaction[a]
	ADE vs control	(95% CI)	ADE vs control	(95% CI)	ADE vs control	(95% CI)	ADE vs control	(95% CI)	
Age									
Younger adults	.30	(.11, .49)	.68	(.49, .88)	.63	(.44, .82)	.86	(.68, 1.05)	.79
Older adults	.23	(.04, .41)	.51	(.33, .69)	.53	(.34, .71)	.77	(.58, .95)	
Education level									
High school diploma or less	.14	(-.22, .50)	.50	(.15, .85)	.78	(.40, 1.15)	.94	(.57, 1.32)	**.007**
Some college	.45	(.16, .74)	.71	(.42, .99)	.71	(.42, .99)	1.15	(.86, 1.44)	
College graduate or associate's degree	.17	(-.01, .36)	.59	(.40, .77)	.57	(.39, .76)	.75	(.57, .94)	
Graduate degree	.44	(.10, .78)	.58	(.24, .92)	.23	(-.09, .55)	.42	(.07, .77)	
Political affiliation									
Democrat	.44	(.26, .61)	.74	(.57, .92)	.78	(.61, .95)	.87	(.70, 1.04)	**.001**
Republican	-.07	(-.33, .19)	.30	(.03, .57)	.20	(-.07, .46)	.44	(.17, .72)	
Independent or Other	.21	(-.08, .51)	.60	(.32, .88)	.43	(.13, .73)	1.08	(.78, 1.39)	

ADE = Average Differential Effect.

[a]*p* for interaction is for Wald tests of the joint significance of the coefficients on all interaction terms. Statistically significant interactions are in bold, p<.05.
