# Supplementary material for: Developing ecolabels to encourage sustainable eating in restaurants: A randomized experiment
Source: PLoS One. 2025 Oct 30;20(10):e0335724. doi: 10.1371/journal.pone.0335724 (PMC12574897; doi:10.1371/journal.pone.0335724)
Supplement: S1 File — (PDF) [file pone.0335724.s006.pdf]

Linear Mixed Effects Regression Results

1) Ecolabel Formats Experiment

1.1) Perceived Message Effectiveness

```
Mixed-effects ML regression                     Number of obs    =  6,507
Group variable: pid                             Number of groups =  2,169
                                                Obs per group:
                                                             min =      3
                                                             avg =    3.0
                                                             max =      3
                                                Wald chi2(4)     = 178.63
Log likelihood = -8195.3993                     Prob > chi2      = 0.0000

------------------------------------------------------------------------------
         pme | Coefficient  Std. err.      z    P>|z|     [95% conf. interval]
-------------+----------------------------------------------------------------
    ecolabel |
    numeric  |   .2634233   .0677021     3.89   0.000     .1307296    .3961171
       text  |   .5756397   .0674675     8.53   0.000      .443406    .7078735
       icon  |   .5952506   .0674289     8.83   0.000     .4630924    .7274088
   texticon  |   .8165142   .0676626    12.07   0.000     .6838979    .9491305
             |
       _cons |   2.354406   .0477341    49.32   0.000     2.260849    2.447963
------------------------------------------------------------------------------

------------------------------------------------------------------------------
  Random-effects parameters  |   Estimate   Std. err.     [95% conf. interval]
-----------------------------+------------------------------------------------
pid: Identity                |
                  var(_cons) |   .8713587   .0302073        .81412    .9326216
-----------------------------+------------------------------------------------
               var(Residual) |   .3594206   .0077174      .3446086    .3748693
------------------------------------------------------------------------------
LR test vs. linear model: chibar2(01) = 3426.43       Prob >= chibar2 = 0.0000
```

1.1.1) Moderation Analysis by age group (younger and older adults)

```
Mixed-effects ML regression                          Number of obs    =  6,507
Group variable: pid                                  Number of groups =  2,169
                                                     Obs per group:
                                                                  min =      3
                                                                  avg =    3.0
                                                                  max =      3
                                                     Wald chi2(9)     = 181.84
Log likelihood = -8193.9198                          Prob > chi2      = 0.0000

------------------------------------------------------------------------------------
                 pme | Coefficient  Std. err.      z    P>|z|     [95% conf. interval]
--------------------+---------------------------------------------------------------
           ecolabel |
           numeric  |   .2271559   .0945919     2.40   0.016     .0417591    .4125526
              text  |   .5270897   .0932847     5.65   0.000      .344255    .7099244
              icon  |   .5113264   .0934941     5.47   0.000     .3280814    .6945715
          texticon  |   .7672496   .0957829     8.01   0.000     .5795186    .9549807
                    |
       1.youngadult |   -.041566   .0955367    -0.44   0.664    -.2288144    .1456825
                    |
ecolabel#youngadult |
         numeric#1  |   .0745791   .1354069     0.55   0.582    -.1908135    .3399718
            text#1  |   .1014971   .1349828     0.75   0.452    -.1630644    .3660586
            icon#1  |   .1733637   .1348817     1.29   0.199    -.0909997    .4377271
        texticon#1  |   .0977298   .1354242     0.72   0.471    -.1676967    .3631562
                    |
              _cons |    2.37409   .0657445    36.11   0.000     2.245233    2.502947
------------------------------------------------------------------------------------

------------------------------------------------------------------------------
  Random-effects parameters  |   Estimate   Std. err.     [95% conf. interval]
-----------------------------+------------------------------------------------
pid: Identity                |
                  var(_cons) |   .8700074   .0301664      .8128466    .9311878
-----------------------------+------------------------------------------------
               var(Residual) |   .3594206   .0077174      .3446086    .3748693
------------------------------------------------------------------------------
LR test vs. linear model: chibar2(01) = 3422.24       Prob >= chibar2 = 0.0000

. testparm i.ecolabel#i.youngadult

 ( 1)  [pme]2.ecolabel#1.youngadult = 0
 ( 2)  [pme]3.ecolabel#1.youngadult = 0
 ( 3)  [pme]4.ecolabel#1.youngadult = 0
 ( 4)  [pme]5.ecolabel#1.youngadult = 0

           chi2(  4) =    1.70
         Prob > chi2 =    0.7906

. margins ecolabel#youngadult

Adjusted predictions                                     Number of obs = 6,507

Expression: Linear prediction, fixed portion, predict()

------------------------------------------------------------------------------
             |            Delta-method
             |     Margin   std. err.      z    P>|z|     [95% conf. interval]
```

ecolabel#youngadult						
control#0	2.37409	.0657445	36.11	0.000	2.245233	2.502947
control#1	2.332524	.0693176	33.65	0.000	2.196664	2.468384
numeric#0	2.601246	.0680096	38.25	0.000	2.46795	2.734542
numeric#1	2.634259	.067694	38.91	0.000	2.501582	2.766937
text#0	2.90118	.0661794	43.84	0.000	2.771471	3.030889
text#1	2.961111	.0686542	43.13	0.000	2.826551	3.095671
icon#0	2.885417	.0664742	43.41	0.000	2.75513	3.015704
icon#1	3.017214	.068169	44.26	0.000	2.883606	3.150823
texticon#0	3.14134	.0696565	45.10	0.000	3.004816	3.277864
texticon#1	3.197504	.0660334	48.42	0.000	3.068081	3.326927

. margins youngadult, dydx(ecolabel)

Conditional marginal effects Number of obs = 6,507

Expression: Linear prediction, fixed portion, predict()
dy/dx wrt: 2.ecolabel 3.ecolabel 4.ecolabel 5.ecolabel

	dy/dx	Delta-method std. err.	z	P>|z|	[95% conf.	interval]
1.ecolabel	(base outcome)					
2.ecolabel youngadult						
0	.2271559	.0945919	2.40	0.016	.0417591	.4125526
1	.301735	.0968886	3.11	0.002	.1118369	.4916331
3.ecolabel youngadult						
0	.5270897	.0932847	5.65	0.000	.344255	.7099244
1	.6285868	.0975619	6.44	0.000	.437369	.8198047
4.ecolabel youngadult						
0	.5113264	.0934941	5.47	0.000	.3280814	.6945715
1	.6846901	.0972211	7.04	0.000	.4941403	.8752399
5.ecolabel youngadult						
0	.7672496	.0957829	8.01	0.000	.5795186	.9549807
1	.8649794	.0957358	9.04	0.000	.6773407	1.052618

Note: dy/dx for factor levels is the discrete change from the base level.

1.1.2) Moderation Analysis by educational attainment

```
mixed pme i.ecolabel##i.educcat || pid: , mle

Performing EM optimization ...

Performing gradient-based optimization:
Iteration 0:  Log likelihood = -8174.5287
Iteration 1:  Log likelihood = -8174.5287

Computing standard errors ...

Mixed-effects ML regression                         Number of obs    =  6,507
Group variable: pid                                 Number of groups =  2,169
                                                    Obs per group:
                                                                 min =      3
                                                                 avg =    3.0
                                                                 max =      3
                                                    Wald chi2(19)    = 224.25
Log likelihood = -8174.5287                         Prob > chi2      = 0.0000

-------------------------------------------------------------------------------
          pme | Coefficient  Std. err.      z    P>|z|     [95% conf. interval]
--------------+----------------------------------------------------------------
     ecolabel |
     numeric  |   .1410714   .1832119     0.77   0.441    -.2180172    .5001601
        text  |   .7763278   .1898936     4.09   0.000     .4041432    1.148513
        icon  |   .5025449   .1773498     2.83   0.005     .1549457     .850144
    texticon  |   .9429945   .1898936     4.97   0.000     .5708098    1.315179
              |
      educcat |
 Some college |  -.1155075   .1661219    -0.70   0.487    -.4411004    .2100854
College gra.. |   .2163801   .1478624     1.46   0.143    -.0734249    .5061851
Graduate de.. |   .3513655    .177933     1.97   0.048     .0026232    .7001079
              |
     ecolabel#|
      educcat |
     numeric #|
 Some college |   .3101154    .235031     1.32   0.187    -.1505369    .7707676
     numeric #|
College gra.. |   .0299204   .2059801     0.15   0.885    -.3737933     .433634
     numeric #|
Graduate de.. |   .2956775   .2520844     1.17   0.241    -.1983989    .7897538
        text #|
 Some college |  -.0699427   .2399991    -0.29   0.771    -.5403322    .4004469
        text #|
College gra.. |  -.2039907   .2119453    -0.96   0.336     -.619396    .2114145
        text #|
Graduate de.. |  -.5449553   .2516374    -2.17   0.030    -1.038156   -.0517551
        icon #|
 Some college |   .2065622   .2293727     0.90   0.368        -.243    .6561244
        icon #|
College gra.. |   .0860866   .2010839     0.43   0.669    -.3080306    .4802038
        icon #|
Graduate de.. |   .0793653   .2478566     0.32   0.749    -.4064248    .5651554
    texticon #|
 Some college |   .2081247   .2397282     0.87   0.385     -.261734    .6779833
    texticon #|
College gra.. |  -.1894896    .211378    -0.90   0.370    -.6037829    .2248037
    texticon #|
Graduate de.. |  -.5265029   .2602302    -2.02   0.043    -1.036545   -.0164611
```

```
             |
       _cons |   2.217262    .131765    16.83   0.000     1.959007    2.475517
------------------------------------------------------------------------------

------------------------------------------------------------------------------
  Random-effects parameters  |   Estimate   Std. err.     [95% conf. interval]
-----------------------------+------------------------------------------------
pid: Identity                |
                  var(_cons) |   .8524666   .0296358      .7963163    .9125762
-----------------------------+------------------------------------------------
               var(Residual) |   .3594206   .0077174      .3446086    .3748693
------------------------------------------------------------------------------
LR test vs. linear model: chibar2(01) = 3367.52       Prob >= chibar2 = 0.0000

. testparm i.ecolabel#i.educcat

 ( 1)  [pme]2.ecolabel#2.educcat = 0
 ( 2)  [pme]2.ecolabel#3.educcat = 0
 ( 3)  [pme]2.ecolabel#4.educcat = 0
 ( 4)  [pme]3.ecolabel#2.educcat = 0
 ( 5)  [pme]3.ecolabel#3.educcat = 0
 ( 6)  [pme]3.ecolabel#4.educcat = 0
 ( 7)  [pme]4.ecolabel#2.educcat = 0
 ( 8)  [pme]4.ecolabel#3.educcat = 0
 ( 9)  [pme]4.ecolabel#4.educcat = 0
 (10)  [pme]5.ecolabel#2.educcat = 0
 (11)  [pme]5.ecolabel#3.educcat = 0
 (12)  [pme]5.ecolabel#4.educcat = 0

           chi2( 12) =   27.25
         Prob > chi2 =    0.0071

. margins ecolabel#educcat

Adjusted predictions                                     Number of obs = 6,507

Expression: Linear prediction, fixed portion, predict()

------------------------------------------------------------------------------
             |            Delta-method
             |     Margin   std. err.      z    P>|z|     [95% conf. interval]
-------------+----------------------------------------------------------------
   ecolabel#|
    educcat |
   control #|
High school..  |   2.217262    .131765    16.83   0.000     1.959007    2.475517
   control #|
 Some college  |   2.101754   .1011655    20.78   0.000     1.903474    2.300035
   control #|
College gra..  |   2.433642   .0670915    36.27   0.000     2.302145    2.565139
   control #|
Graduate de..  |   2.568627   .1195748    21.48   0.000     2.334265     2.80299
   numeric #|
High school..  |   2.358333   .1272971    18.53   0.000     2.108836    2.607831
   numeric #|
 Some college  |   2.552941    .106951    23.87   0.000     2.343321    2.762561
   numeric #|
College gra..  |   2.604634   .0660301    39.45   0.000     2.475217     2.73405
   numeric #|
```

```
Graduate de..  |   3.005376   .1252271    24.00   0.000     2.759936    3.250817
         text #|
High school..  |    2.99359    .136739    21.89   0.000     2.725586    3.261593
         text #|
 Some college  |    2.80814   .1063274    26.41   0.000     2.599742    3.016537
         text #|
College gra..  |   3.005979   .0660301    45.52   0.000     2.876563    3.135396
         text #|
Graduate de..  |        2.8    .113858    24.59   0.000     2.576842    3.023158
         icon #|
High school..  |   2.719807   .1187052    22.91   0.000     2.487149    2.952465
         icon #|
 Some college  |   2.810861     .10452    26.89   0.000     2.606006    3.015717
         icon #|
College gra..  |   3.022273   .0669367    45.15   0.000      2.89108    3.153467
         icon #|
Graduate de..  |   3.150538   .1252271    25.16   0.000     2.905097    3.395978
     texticon #|
High school..  |   3.160256    .136739    23.11   0.000     2.892253     3.42826
     texticon #|
 Some college  |   3.252874   .1057145    30.77   0.000     3.045677     3.46007
     texticon #|
College gra..  |   3.187147   .0641857    49.66   0.000     3.061345    3.312949
     texticon #|
Graduate de..  |   2.985119    .131765    22.65   0.000     2.726864    3.243374
--------------------------------------------------------------------------------

. margins educcat, dydx(ecolabel)

Conditional marginal effects                            Number of obs = 6,507

Expression: Linear prediction, fixed portion, predict()
dy/dx wrt:  2.ecolabel 3.ecolabel 4.ecolabel 5.ecolabel

--------------------------------------------------------------------------------
               |            Delta-method
               |      dy/dx   std. err.      z    P>|z|     [95% conf. interval]
---------------+----------------------------------------------------------------
1.ecolabel     |  (base outcome)
---------------+----------------------------------------------------------------
2.ecolabel     |
       educcat |
High school..  |   .1410714   .1832119     0.77   0.441    -.2180172    .5001601
 Some college  |   .4511868   .1472174     3.06   0.002     .1626459    .7397276
College gra..  |   .1709918   .0941341     1.82   0.069    -.0135077    .3554913
Graduate de..  |   .4367489   .1731472     2.52   0.012     .0973865    .7761113
---------------+----------------------------------------------------------------
3.ecolabel     |
       educcat |
High school..  |   .7763278   .1898936     4.09   0.000     .4041432    1.148513
 Some college  |   .7063851    .146765     4.81   0.000      .418731    .9940392
College gra..  |   .5723371   .0941341     6.08   0.000     .3878376    .7568366
Graduate de..  |   .2313725   .1651114     1.40   0.161    -.0922399     .554985
---------------+----------------------------------------------------------------
4.ecolabel     |
       educcat |
High school..  |   .5025449   .1773498     2.83   0.005     .1549457     .850144
 Some college  |    .709107   .1454609     4.87   0.000     .4240089    .9942052
College gra..  |   .5886315   .0947723     6.21   0.000     .4028812    .7743817
Graduate de..  |   .5819102   .1731472     3.36   0.001     .2425478    .9212726
---------------+----------------------------------------------------------------
```

5.ecolabel						
educcat						
High school..	.9429945	.1898936	4.97	0.000	.5708098	1.315179
Some college	1.151119	.1463216	7.87	0.000	.8643341	1.437904
College gra..	.7535049	.0928497	8.12	0.000	.5715228	.935487
Graduate de..	.4164916	.177933	2.34	0.019	.0677493	.7652339

Note: dy/dx for factor levels is the discrete change from the base level.

1.1.3) Moderation Analysis by political party affiliation

```
. mixed pme i.ecolabel##i.partyidcat2 || pid: , mle

Performing EM optimization ...

Performing gradient-based optimization:
Iteration 0:  Log likelihood = -8164.7923
Iteration 1:  Log likelihood = -8164.7923

Computing standard errors ...

Mixed-effects ML regression                         Number of obs    =  6,507
Group variable: pid                                 Number of groups =  2,169
                                                    Obs per group:
                                                                 min =      3
                                                                 avg =    3.0
                                                                 max =      3
                                                    Wald chi2(14)    = 245.83
Log likelihood = -8164.7923                         Prob > chi2      = 0.0000

------------------------------------------------------------------------------
         pme | Coefficient  Std. err.      z    P>|z|     [95% conf. interval]
-------------+----------------------------------------------------------------
    ecolabel |
    numeric  |   .4372826   .0897361     4.87   0.000     .2614032    .6131621
       text  |    .777883   .0880681     8.83   0.000     .6052728    .9504933
       icon  |   .7443433   .0901377     8.26   0.000     .5676767    .9210098
   texticon  |   .8717416    .086909    10.03   0.000     .7014031     1.04208
             |
 partyidcat2 |
 Republican  |   .1640147    .114429     1.43   0.152     -.060262    .3882914
Independent..  |  -.1836043   .1240494    -1.48   0.139    -.4267366    .0595279
             |
    ecolabel#|
 partyidcat2 |
    numeric #|
 Republican  |  -.5071239   .1602562    -3.16   0.002    -.8212202   -.1930276
    numeric #|
Independent..  |  -.2244312   .1764461    -1.27   0.203    -.5702593    .1213968
       text #|
 Republican  |  -.5810577   .1615858    -3.60   0.000    -.8977599   -.2643554
       text #|
Independent..  |  -.3487164    .176839    -1.97   0.049    -.6953144   -.0021184
       icon #|
 Republican  |  -.4447131   .1632696    -2.72   0.006    -.7647157   -.1247106
       icon #|
Independent..  |  -.1443433   .1696261    -0.85   0.395    -.4768043    .1881178
   texticon #|
 Republican  |  -.4281518   .1652846    -2.59   0.010    -.7521037      -.1042
   texticon #|
Independent..  |   .2104806   .1785278     1.18   0.238    -.1394275    .5603888
             |
       _cons |   2.350271    .062586    37.55   0.000     2.227605    2.472937
------------------------------------------------------------------------------

------------------------------------------------------------------------------
  Random-effects parameters  |   Estimate   Std. err.     [95% conf. interval]
-----------------------------+------------------------------------------------
pid: Identity                |
                  var(_cons) |   .8437768   .0293729      .7881271     .903356
```

```
-----------------------------+------------------------------------------------
              var(Residual) |   .3594206   .0077174      .3446086    .3748693
------------------------------------------------------------------------------
LR test vs. linear model: chibar2(01) = 3340.16       Prob >= chibar2 = 0.0000


. testparm i.ecolabel#i.partyidcat2

 ( 1)  [pme]2.ecolabel#2.partyidcat2 = 0
 ( 2)  [pme]2.ecolabel#3.partyidcat2 = 0
 ( 3)  [pme]3.ecolabel#2.partyidcat2 = 0
 ( 4)  [pme]3.ecolabel#3.partyidcat2 = 0
 ( 5)  [pme]4.ecolabel#2.partyidcat2 = 0
 ( 6)  [pme]4.ecolabel#3.partyidcat2 = 0
 ( 7)  [pme]5.ecolabel#2.partyidcat2 = 0
 ( 8)  [pme]5.ecolabel#3.partyidcat2 = 0

           chi2(  8) =   25.52
         Prob > chi2 =    0.0013


. margins ecolabel#partyidcat2

Adjusted predictions                                    Number of obs = 6,507

Expression: Linear prediction, fixed portion, predict()

--------------------------------------------------------------------------------
               |            Delta-method
               |     Margin   std. err.      z    P>|z|     [95% conf. interval]
---------------+----------------------------------------------------------------
      ecolabel#|
   partyidcat2 |
      control #|
     Democrat  |   2.350271    .062586    37.55   0.000     2.227605    2.472937
      control #|
   Republican  |   2.514286   .0957966    26.25   0.000     2.326528    2.702044
      control #|
Independent..  |   2.166667   .1071038    20.23   0.000     1.956747    2.376586
      numeric #|
     Democrat  |   2.787554   .0643083    43.35   0.000     2.661512    2.913596
      numeric #|
   Republican  |   2.444444   .0919374    26.59   0.000      2.26425    2.624638
      numeric #|
Independent..  |   2.379518   .1077471    22.08   0.000     2.168338    2.590699
text#Democrat  |   3.128154   .0619595    50.49   0.000     3.006716    3.249592
         text #|
   Republican  |   2.711111   .0957966    28.30   0.000     2.523353    2.898869
         text #|
Independent..  |   2.595833   .1097488    23.65   0.000      2.38073    2.810937
icon#Democrat  |   3.094614   .0648675    47.71   0.000     2.967476    3.221752
         icon #|
   Republican  |   2.813916   .0967222    29.09   0.000     2.624344    3.003488
         icon #|
Independent..  |   2.766667   .0957966    28.88   0.000     2.578909    2.954425
     texticon #|
     Democrat  |   3.222013   .0603006    53.43   0.000     3.103826      3.3402
     texticon #|
   Republican  |   2.957875   .1029021    28.74   0.000     2.756191     3.15956
     texticon #|
Independent..  |   3.248889   .1133481    28.66   0.000     3.026731    3.471047
```

```
--------------------------------------------------------------------------------


. margins partyidcat2, dydx(ecolabel)

Conditional marginal effects                                Number of obs = 6,507

Expression: Linear prediction, fixed portion, predict()
dy/dx wrt:  2.ecolabel 3.ecolabel 4.ecolabel 5.ecolabel

--------------------------------------------------------------------------------
               |            Delta-method
               |      dy/dx   std. err.      z    P>|z|     [95% conf. interval]
---------------+----------------------------------------------------------------
1.ecolabel     |  (base outcome)
---------------+----------------------------------------------------------------
2.ecolabel     |
   partyidcat2 |
     Democrat  |   .4372826   .0897361     4.87   0.000     .2614032    .6131621
   Republican  |  -.0698413    .132776    -0.53   0.599    -.3300775     .190395
Independent..  |   .2128514   .1519233     1.40   0.161    -.0849127    .5106155
---------------+----------------------------------------------------------------
3.ecolabel     |
   partyidcat2 |
     Democrat  |    .777883   .0880681     8.83   0.000     .6052728    .9504933
   Republican  |   .1968254   .1354768     1.45   0.146    -.0687043    .4623551
Independent..  |   .4291667   .1533494     2.80   0.005     .1286074    .7297259
---------------+----------------------------------------------------------------
4.ecolabel     |
   partyidcat2 |
     Democrat  |   .7443433   .0901377     8.26   0.000     .5676767    .9210098
   Republican  |   .2996301   .1361329     2.20   0.028     .0328145    .5664457
Independent..  |         .6   .1436949     4.18   0.000     .3183632    .8816368
---------------+----------------------------------------------------------------
5.ecolabel     |
   partyidcat2 |
     Democrat  |   .8717416    .086909    10.03   0.000     .7014031     1.04208
   Republican  |   .4435897    .140591     3.16   0.002     .1680365     .719143
Independent..  |   1.082222   .1559456     6.94   0.000     .7765745     1.38787
--------------------------------------------------------------------------------
Note: dy/dx for factor levels is the discrete change from the base level.
```

1.2) Thinking about environmental impacts of foods

```
Mixed-effects ML regression                         Number of obs    =  6,507
Group variable: pid                                 Number of groups =  2,169
                                                    Obs per group:
                                                                 min =      3
                                                                 avg =    3.0
                                                                 max =      3
                                                    Wald chi2(4)     = 546.14
Log likelihood = -8979.2717                         Prob > chi2      = 0.0000

------------------------------------------------------------------------------
        elab | Coefficient  Std. err.      z    P>|z|     [95% conf. interval]
-------------+----------------------------------------------------------------
    ecolabel |
    numeric  |   1.095073   .0695223    15.75   0.000     .9588113    1.231334
       text  |   1.267944   .0692814    18.30   0.000     1.132155    1.403733
       icon  |   1.115898   .0692418    16.12   0.000     .9801868     1.25161
   texticon  |    1.46954   .0694818    21.15   0.000     1.333358    1.605722
```

```
             |
       _cons |   1.842912   .0490174    37.60   0.000     1.746839    1.938984
------------------------------------------------------------------------------

------------------------------------------------------------------------------
  Random-effects parameters  |   Estimate   Std. err.     [95% conf. interval]
-----------------------------+------------------------------------------------
pid: Identity                |
                  var(_cons) |   .8777175   .0319407      .8172952    .9426068
-----------------------------+------------------------------------------------
               var(Residual) |    .502382   .0107871      .4816784    .5239755
------------------------------------------------------------------------------
LR test vs. linear model: chibar2(01) = 2603.79       Prob >= chibar2 = 0.0000
```

1.3) Anticipated social interactions

Mixed-effects ML regression
Group variable: pid

Number of obs = 6,507
Number of groups = 2,169
Obs per group:
min = 3
avg = 3.0
max = 3
Wald chi2(4) = 48.18
Prob > chi2 = 0.0000

Log likelihood = -8333.1809

talk	Coefficient	Std. err.	z	P>|z|	[95% conf.	interval]
ecolabel						
numeric	.4502183	.0769019	5.85	0.000	.2994933	.6009433
text	.2945288	.0766354	3.84	0.000	.1443263	.4447314
icon	.0911421	.0765916	1.19	0.234	-.0589745	.2412588
texticon	.3635153	.076857	4.73	0.000	.2128783	.5141523
_cons	1.918774	.0542205	35.39	0.000	1.812504	2.025044

Random-effects parameters	Estimate	Std. err.	[95% conf.	interval]
pid: Identity				
var(_cons)	1.166448	.038908	1.092629	1.245254
var(Residual)	.3371753	.0072398	.32328	.3516679

LR test vs. linear model: chibar2(01) = 4453.76 Prob >= chibar2 = 0.0000

1.4) Attention

```
Mixed-effects ML regression                     Number of obs    =  6,506
Group variable: pid                             Number of groups =  2,169
                                                Obs per group:
                                                             min =      2
                                                             avg =    3.0
                                                             max =      3
                                                Wald chi2(4)     =  37.34
Log likelihood = -8873.6019                     Prob > chi2      = 0.0000

------------------------------------------------------------------------------
        attn | Coefficient  Std. err.      z    P>|z|     [95% conf. interval]
-------------+----------------------------------------------------------------
    ecolabel |
    numeric  |   -.052829   .0597692    -0.88   0.377    -.1699746    .0643165
       text  |  -.0039527   .0595621    -0.07   0.947    -.1206922    .1127868
       icon  |  -.1725936   .0595317    -2.90   0.004    -.2892736   -.0559137
   texticon  |   .1853677   .0597344     3.10   0.002     .0682905    .3024449
             |
       _cons |   3.390038   .0421409    80.45   0.000     3.307444    3.472633
------------------------------------------------------------------------------

------------------------------------------------------------------------------
  Random-effects parameters  |   Estimate   Std. err.     [95% conf. interval]
-----------------------------+------------------------------------------------
pid: Identity                |
                  var(_cons) |    .586864   .0237938      .5420338    .6354021
-----------------------------+------------------------------------------------
               var(Residual) |   .5569003   .0119588      .5339478    .5808393
------------------------------------------------------------------------------
LR test vs. linear model: chibar2(01) = 1590.27       Prob >= chibar2 = 0.0000
```

1.5) Believability

```
Mixed-effects ML regression                          Number of obs    =  6,507
Group variable: pid                                  Number of groups =  2,169
                                                     Obs per group:
                                                                  min =      3
                                                                  avg =    3.0
                                                                  max =      3
                                                     Wald chi2(4)     = 130.28
Log likelihood = -8616.2908                          Prob > chi2      = 0.0000

------------------------------------------------------------------------------
     believe | Coefficient  Std. err.      z    P>|z|     [95% conf. interval]
-------------+----------------------------------------------------------------
    ecolabel |
    numeric  |  -.6083846   .0631562    -9.63   0.000    -.7321684   -.4846008
       text  |  -.6261749   .0629372    -9.95   0.000    -.7495296   -.5028202
       icon  |  -.4760287   .0629013    -7.57   0.000    -.5993129   -.3527444
   texticon  |  -.4677903   .0631193    -7.41   0.000    -.5915019   -.3440788
             |
       _cons |   3.678927   .0445289    82.62   0.000     3.591652    3.766202
------------------------------------------------------------------------------

------------------------------------------------------------------------------
  Random-effects parameters  |   Estimate   Std. err.     [95% conf. interval]
-----------------------------+------------------------------------------------
pid: Identity                |
                  var(_cons) |    .706593   .0264045      .6566908    .7602872
-----------------------------+------------------------------------------------
               var(Residual) |   .4678039   .0100446      .4485253    .4879111
------------------------------------------------------------------------------
LR test vs. linear model: chibar2(01) = 2279.51       Prob >= chibar2 = 0.0000
```

2) Ecolabel Text and Icon Variations

2.1) Topic Variations

```
Mixed-effects ML regression                         Number of obs    =  13,014
Group variable: pid                                 Number of groups =   2,169
                                                    Obs per group:
                                                                 min =       6
                                                                 avg =     6.0
                                                                 max =       6
                                                    Wald chi2(5)     = 1441.63
Log likelihood = -16951.856                         Prob > chi2      =  0.0000

------------------------------------------------------------------------------
         enc | Coefficient  Std. err.      z    P>|z|     [95% conf. interval]
-------------+----------------------------------------------------------------
  lowclimate |
     earthfr |   .2697095   .0220924    12.21   0.000     .2264093    .3130098
 sust.choice |   .3748271   .0220924    16.97   0.000     .3315268    .4181274
    envirofr |   .4011065   .0220924    18.16   0.000     .3578062    .4444068
   climatefr |   .1272476   .0220924     5.76   0.000     .0839473    .1705478
   lowcarbon |  -.2982941   .0220924   -13.50   0.000    -.3415944   -.2549939
             |
       _cons |   2.854311   .0257124   111.01   0.000     2.803915    2.904706
------------------------------------------------------------------------------

------------------------------------------------------------------------------
  Random-effects parameters  |   Estimate   Std. err.     [95% conf. interval]
-----------------------------+------------------------------------------------
pid: Identity                |
                  var(_cons) |    .904674   .0301738      .8474259    .9657895
-----------------------------+------------------------------------------------
               var(Residual) |   .5293152   .0071881      .5154126    .5435928
------------------------------------------------------------------------------
LR test vs. linear model: chibar2(01) = 7719.44       Prob >= chibar2 = 0.0000
```

2.2) Icon Variations

```
Mixed-effects ML regression                     Number of obs    =   8,676
Group variable: pid                             Number of groups =   2,169
                                                Obs per group:
                                                             min =       4
                                                             avg =     4.0
                                                             max =       4
                                                Wald chi2(3)     = 1673.28
Log likelihood = -12413.104                     Prob > chi2      =  0.0000

------------------------------------------------------------------------------
         enc | Coefficient  Std. err.      z    P>|z|     [95% conf. interval]
-------------+----------------------------------------------------------------
      leaves |
       globe |   .0359613   .0253227     1.42   0.156    -.0136702    .0855928
   checkmark |  -.5371139   .0253227   -21.21   0.000    -.5867454   -.4874824
    coolfood |  -.8307976   .0253227   -32.81   0.000    -.8804291   -.7811661
             |
       _cons |    3.07976   .0248418   123.97   0.000     3.031071    3.128449
------------------------------------------------------------------------------

------------------------------------------------------------------------------
  Random-effects parameters  |   Estimate   Std. err.     [95% conf. interval]
-----------------------------+------------------------------------------------
pid: Identity                |
                  var(_cons) |   .6431009   .0249941      .5959326    .6940025
-----------------------------+------------------------------------------------
               var(Residual) |   .6954219   .0121919       .671932     .719733
------------------------------------------------------------------------------
LR test vs. linear model: chibar2(01) = 2324.84       Prob >= chibar2 = 0.0000
```
